# Increased Amygdala-Paracentral Lobule/Precuneus Functional Connectivity Associated With Patients With Mood Disorder and Suicidal Behavior

**DOI:** 10.3389/fnhum.2020.585664

**Published:** 2021-01-15

**Authors:** Ran Zhang, Luheng Zhang, Shengnan Wei, Pengshuo Wang, Xiaowei Jiang, Yanqing Tang, Fei Wang

**Affiliations:** ^1^Department of Psychiatry, First Affiliated Hospital, China Medical University, Shenyang, China; ^2^Brain Function Research Section, First Affiliated Hospital, China Medical University, Shenyang, China; ^3^Department of Radiology, First Affiliated Hospital, China Medical University, Shenyang, China; ^4^Department of Geriatric Medicine, First Affiliated Hospital, China Medical University, Shenyang, China

**Keywords:** mood disorder, suicidal behavior, fMRI—functional magnetic resonance imaging, functional connectivity, amygdala

## Abstract

Mood disorder patients have greater suicide risk than members of the general population, but how suicidal behavior relates to brain functions has not been fully elucidated. This study investigated how functional connectivity (FC) values between the right/left amygdala and the whole brain relate to suicidal behavior in patients with mood disorder. The participants in this study were 100 mood disorder patients with suicidal behavior (SB group), 120 mood disorder patients with non-suicidal behavior (NSB group), and 138 age- and gender-matched healthy controls (HC group). Whole-brain FC values among the three groups were compared using an analysis of covariance (ANCOVA). Compared to the NSB and HC groups, increased FC values in the right amygdala-bilateral paracentral lobule/precuneus circuit were observed in the SB group (Bonferroni-corrected, *p* < 0.017). The FC values in the NSB group did not differ significantly from those in the HC group (Bonferroni-corrected, *p* > 0.017). Moreover, there were no significant differences in FC values between mood disorder patients with suicide attempt (SA group) and mood disorder patients with suicidal ideation (SI group), while the FC values between the right amygdala and bilateral paracentral lobule/precuneus in the SA group were higher than the mean in the SI group. These findings suggest that right amygdala-paracentral lobule/precuneus dysfunction has an important role in patients with mood disorder and suicidal behavior.

## Introduction

Suicide is a worldwide public health problem. Annually, more than 800 000 people die by suicide according to the World Health Organization (Health, 2017), making suicide the third leading cause of death in the age group of 15–44 years (Bertolote and Fleischmann, 2015). Mood disorders, including major depressive disorder (MDD) and bipolar disorder (BD), are both are risk factors for suicide (Bachmann, 2018). High rates of suicide in patients with a mood disorder, who account for 30.2% of cases (Bertolote et al., 2004), suggest that greater emphasis should also be placed on the risk identification and prevention of suicide. Several studies have indicated that a prior suicide attempt and suicidal ideation are important predictive factors for suicide in the population with mood disorders (Brown et al., 2000; Kuo et al., 2001; Klonsky et al., 2016). For every suicide, there are many more people who attempt suicide and have suicidal ideation every year (World Health Organization, 2014). To improve the availability and quality of biomarkers for effective suicide prevention, many studies employing various methods have been conducted to analyze suicidal behavior in mood disorder patients.

Recent magnetic resonance imaging (MRI) studies have suggested that structural and functional changes in the brain may be involved in suicidal behavior in patients with mood disorders. In patients with MDD, several previous functional MRI studies found that MDD combined with suicide attempts and suicidal ideation was associated with altered brain function. A recent functional MRI experiment revealed a significant correlation between suicide and the functional connectivity (FC) of the right amygdala with the right parahippocampal area in subjects with MDD (Kang et al., 2017). Moreover, in prior studies, an association between the frontal cortex and suicide attempts was reported in currently depressed female (Reisch et al., 2010) and male (Jollant et al., 2010) patients. Compared to non-attempters, adolescent suicide attempters with MDD exhibited differential activation of the right anterior cingulate gyrus during response inhibition (Pan et al., 2011). A study on MDD patients with suicidal ideation also showed that striatal-anterior cortical midline structure (CMS) circuitry (Marchand et al., 2012) likely plays an important role in MDD and suicidal ideation. Furthermore, a few studies suggested that brain regions, including the fronto-limbic (Du et al., 2017) circuit and basal ganglia (Kang et al., 2017), may be involved in brain functions that are relevant for both suicide and depression. In patients with BD, a number of studies demonstrated findings involving the structure or function of CMS circuitry (Marchand et al., 2011), the frontal cortex (Duarte et al., 2017), and fronto-limbic structure (Johnston et al., 2017). Interestingly, previous functional MRI studies about mood disorders (MDD and BD) have also shown that those with suicidal ideation and attempt histories exhibit distinct fronto-striatal function (Minzenberg et al., 2015). Furthermore, structural studies and diffusion tensor imaging (DTI) studies reported that, compared to patients who had no history of suicide attempts or suicidal ideation, MDD, BD, or mood disorder patients with a history of suicide attempt or ideation show gray matter abnormalities and white matter integrity in the amygdala (Monkul et al., 2007; Gifuni et al., 2016), corpus callosum (Matsuo et al., 2010; Cyprien et al., 2016), basal ganglia (Ahearn et al., 2001), paralimbic cortex (the precuneus/posterior cingulate cortex) (Vanyukov et al., 2016), parietal lobe (Chen et al., 2015), temporal gyrus (Pan et al., 2015), hippocampus (Colle et al., 2015), fronto-thalamic loops (Jia et al., 2014), fronto-limbic loops (Monkul et al., 2007) and white matter hyperintensities (Ehrlich et al., 2004; Pompili et al., 2007; Grangeon et al., 2010; Serafini et al., 2011). These findings of prior studies suggest that the brain structures and functional impairments related to emotional and cognitive processing increase the risk of suicide in mood disorder patients with suicidal behavior and show similarities in relevant regions of the brain. Unfortunately, it has not been determined how disruptions in emotion- and cognition-based regions contribute to suicidal behavior in mood disorder patients.

The amygdala, which consists of paired structures located in each hemisphere of the brain, is an important region associated with emotional and cognitive processing (Strobel et al., 2014; Janak and Tye, 2015; Yang and Wang, 2017). Additionally, renewed studies suggest that abnormalities of emotion processing is may serve as a marker for suicidality (Ai et al., 2018; Villa et al., 2018). Proteomic studies on post-mortem brains noted nine proteins that changed in both the amygdala and the cortex (Kekesi et al., 2012). Additionally, a number of neuroimaging studies utilizing structural MRI (Killgore et al., 2009; Tamburo et al., 2009), functional MRI (Arnone et al., 2012; Price and Drevets, 2012), and imaging genetics approaches (Heinrich et al., 2013) have reported that the amygdala plays an important role in mood disorders. Research investigating the neural circuitry connecting the amygdala and whole brain may therefore help to elucidate important neuroimaging changes associated with suicidal behavior in mood disorder patients. Therefore, we selected the amygdala as the region of interest to explore the neuroimaging basis of pre-disposition to suicide in mood disorder patients.

In this study, we performed an FC analysis between the right and left amygdala to investigate the changes in FC values between the amygdala and whole brain in patients with suicidal behavior and mood disorder. Our hypotheses were (1) that compared to healthy controls (HCs) and mood disorder patients without suicidal behavior, patients with suicidal behavior and mood disorder show diagnosis-specific alterations and (2) that the FC values associated with the mood disorder patients with a suicide attempt will differ from those of mood disorder patients with suicidal ideation.

## Materials and Methods

### Subjects

A total of 220 patients with mood disorder (15–50 years of age) between 2012 and 2019 were recruited from the inpatient department of the Shenyang Mental Health Center and the outpatient clinic of the Department of Psychiatry of the First Affiliated Hospital of China Medical University, Shenyang, China. The inclusion criterion was whether the participants met the structured clinical interview for DSM-IV Axis I disorders (SCID-I) diagnostic criteria for mood disorder (MDD, BD type I, or BD type II). The exclusion criteria were (1) no history of other Axis I mental disorders; (2) no history of major somatic disease, especially diseases that may be related to changes in brain tissue, such as hypertension or diabetes; (3) no history of abnormalities of the nervous system, including major head trauma, epilepsy, cerebrovascular disease, brain tumors, or neurodegenerative diseases; and (4) no contraindications for MRI.

In addition, 138 healthy volunteers (15–50 years of age) with no history of suicide attempts or suicidal ideation, no current Axis I disorder, and no history of Axis I disorders in their first-degree relatives according to a detailed family history were recruited from advertisements. The other exclusion criteria were the same as for the participants with mood disorders.

All participants provided written informed consent after receiving detailed descriptions of the study. This study was approved by the Institutional Review Board of China Medical University.

### Clinical Evaluation

A suicide attempt was defined as one or more self-directed injurious acts with a variable degree of intent to end one's own life. Suicidal ideation severity was assessed using the 19-item Beck Scale for Suicide Ideation (Beck et al., 1979). A trained psychiatrist assessed the lifetime suicide histories of the patients by reviewing their medical records. The patients were classified as having a positive suicidal history if they reported suicide attempts and suicidal ideation. Two hundred twenty patients with mood disorder were divided into the suicidal behavior group (SB group) and the non-suicidal behavior group (NSB group). Patients in the SB group were also divided into the suicide attempt group (SA group) and the suicidal ideation group (SI group).

Clinical symptoms were measured using the Hamilton Depression Rating Scale (HAMD), the Hamilton Anxiety Rating Scale (HAMA), and the Young Mania Rating Scale (YMRS).

### Image Acquisition and Processing

MRI scans were performed on a 3.0T GE Sigma system with a standard 8-channel head coil at the First Affiliated Hospital of China Medical University, Shenyang, China. Head motion was minimized with foam pads. For the purposes of this study, we define the resting state as the absence of prescribed cognitive tasks. Therefore, each subject was instructed to remain awake, close their eyes, relax, and minimize thoughts during scanning. The resting-state functional sequence was as follows: TR, 2,000 ms; TE, 30 ms; flip angle, 90°; field of view (FOV), 240 × 240 mm^2^; matrix, 64 × 64; slice thickness, 3 mm without a gap; slice, 35; and voxel size, 3 mm^3^.

Acquired images were processed using the Statistical Parametric Mapping 12 (SPM12, http://www.fil.ion.ucl.ac.uk/spm) and the Data Processing Assistant for R-fMRI (DPARSF; http://www.restfmri.net/forum/DPARSF) toolkits (Chao-Gan and Yu-Feng, 2010). The first 10 time points were discarded due to magnetic saturation effects. The remaining images were first corrected for within-scan acquisition time differences among slices and then realigned to the first volume to correct for head motion. Images from participants with excessive motion (>3 mm of motion and/or 3° rotation) were excluded. Images were normalized to the standard EPI template in Montreal Neurological Institute (MNI) space and resampled to 3 × 3 × 3 mm^3^. Spatial smoothing was performed with a 6-mm full-width at half maximum (FWHM) Gaussian filter. At this stage, linear detrending and temporal bandpass (0.01–0.08 Hz) filtering were performed to remove low-frequency drifts and physiological high-frequency noise. Linear regression of head motion (Friston 24-parameter model was used) (Friston et al., 1996), white matter signals and cerebral spinal fluid signals were performed to remove the effects of the nuisance covariates. Additionally, the global signal also was regressed out in this study because it remains a popular denoising method in reducing the impact of motion artifacts, particularly those resulting from participant head motion (Murphy et al., 2009; Power et al., 2017). Finally, a “scrubbing” approach using Cubic Spline Interpolation was conducted to reduce the impact of motion artifacts (Power et al., 2012, 2013). Framewise displacement (FD) was computed based on their realignment parameters to quantify head motion (Power et al., 2019). The mean FD was calculated by using the average of the volume-based FD time series. Subjects with an FD power of > 0.2 mm were eliminated, as well as uncensored data remaining <125 volumes (Power et al., 2014). Thus, two hundred and one patients, among which 88 have suicidal behavior, and 121 healthy controls were included in the final data analysis. Detailed information for the demographic and clinical data of subjects were performed on Table 1.

**Table 1:** Clinical and demographic characteristic of all patients.

**Characteristic**	**SB (*****N*** **=** **88)**		**NSB (*****N*** **=** **113)**		**HC (*****N*** **=** **121)**		**Analysis**	
	**Mean**	**SD**	**Mean**	**SD**	**Mean**	**SD**		***P***
Age (years)^a^	26.16	9.55	28.19	9.45	28.50	9.54	1.71	0.18
Education (years)^a^	12.65	2.73	12.66	3.26	13.40	2.68	2.50	0.08
Mean FD ^a^	0.09	0.04	0.10	0.04	0.10	0.03	0.77	0.47
HAMD^b^	23.67	6.91	20.71	7.00	–	–	2.99	0.003^*^
HAMA^b^	21.69	8.26	19.72	8.43	–	–	1.66	0.10
	***N***	**%**	***N***	**%**	***N***	**%**		***P***
Gender (male)^c^	19	21.59%	32	28.32%	42	34.71%	4.30	0.12
Mediation (yes)^c^	59	67.05%	72	63.72%	–	–	0.17	0.77

*SB, mood disorder with suicidal behavior; NSB, mood disorder without suicidal behavior; HC, healthy controls; FD, Framewise displacement; HAMD, Hamilton depression rating score; HAMA, Hamilton anxiety rating score*.

a *One-way ANOVA was used; Data are given as the mean and standard deviation*.

b *Student's t-statistic was used; Data are given as the mean and standard deviation*.

c *Chi-squared test was used; Data are given as frequencies and percentage*.

* *Significant at P < 0.05*.

### Functional Connectivity

Using the WFU PickAtlas Tool (http://www.fmri.wfubmc.edu/download.html), two regions of interest (ROIs) based on the Anatomical Automatic Labeling (AAL) atlas located in the left amygdala and the right amygdala were defined as seeds to voxel analysis, resampling to 3 × 3 × 3 mm^3^. Detailed information for seed ROIs were performed on Table 2.

**Table 2:** Seed ROIs to the ROI-voxel analysis.

**Seeds**	**MNI (x, y, z)**	**k**	**AAL**
Left amygdala	−24, 0, −21	64	Amygdala_L
Right amygdala	21, −1, −22	75	Amygdala_R

*MNI, Montreal Neurological Institute coordinate system; AAL, Automated Anatomical Labeling*.

The BOLD time series of the voxels within the ROIs were averaged to generate the reference time series for the ROIs. A voxelwise FC analysis of ROIs was used. For each participant, correlation analysis was carried out between the seed ROIs and the remaining voxels in the whole brain in a voxelwise manner using the Resting-State fMRI Data Analysis Toolkit (REST, http://www.restfmri.net). The calculated correlation coefficients in each map were transformed to *z*-values using Fisher's r-to-z transformation for further statistical testing.

### Statistical Analysis

Continuous variables are presented as the mean and standard deviation (SD), and categorical variables are summarized as frequencies (n) and proportions (%). We first performed a one-way analysis of covariance (ANCOVA) between FC values of the groups (SB vs. NSB vs. HC) in order to detect differences in left amygdala-whole brain connectivity. Mean FD, age and gender were used as covariates. The significant voxel-level threshold was set at *p* < 0.001 after Gaussian Random Fields (GRF)-corrected (threshold cluster size > 36 voxels). The FC values of brain regions showing significant group differences were extracted, and then *post hoc* comparisons were applied to correct for multiple comparisons. Using a Bonferroni correction, a *p*-value of < 0.017 (α = 0.05/3 = 0.017) will be considered statistically significant. Furthermore, we conducted an additional analysis to compare extracted FC values differences between the SA and SI groups. Finally, to evaluate the correlation between clinical symptoms and FC values, the correlations analytic method was applied to the FC values of the significantly different regions. The same analysis was applied in the right amygdala-whole brain connectivity.

## Results

### Demographics and Clinical Characteristics

Table 1 shows demographics and clinical characteristics for all participants. There were no significant differences between the SB and NSB groups with respect to state of mediation and HAMA scores. The SB group had higher HAMD scores (*t* = 2.99, *p* = 0.003 < 0.05) than the NSB group. Furthermore, no significant differences in gender, age, educational level and mean FD were observed between the SB, NSB and HC groups.

### Comparison of Amygdala-Whole Brain Connectivity Among SB, NSB and HC

**Figure 1:**
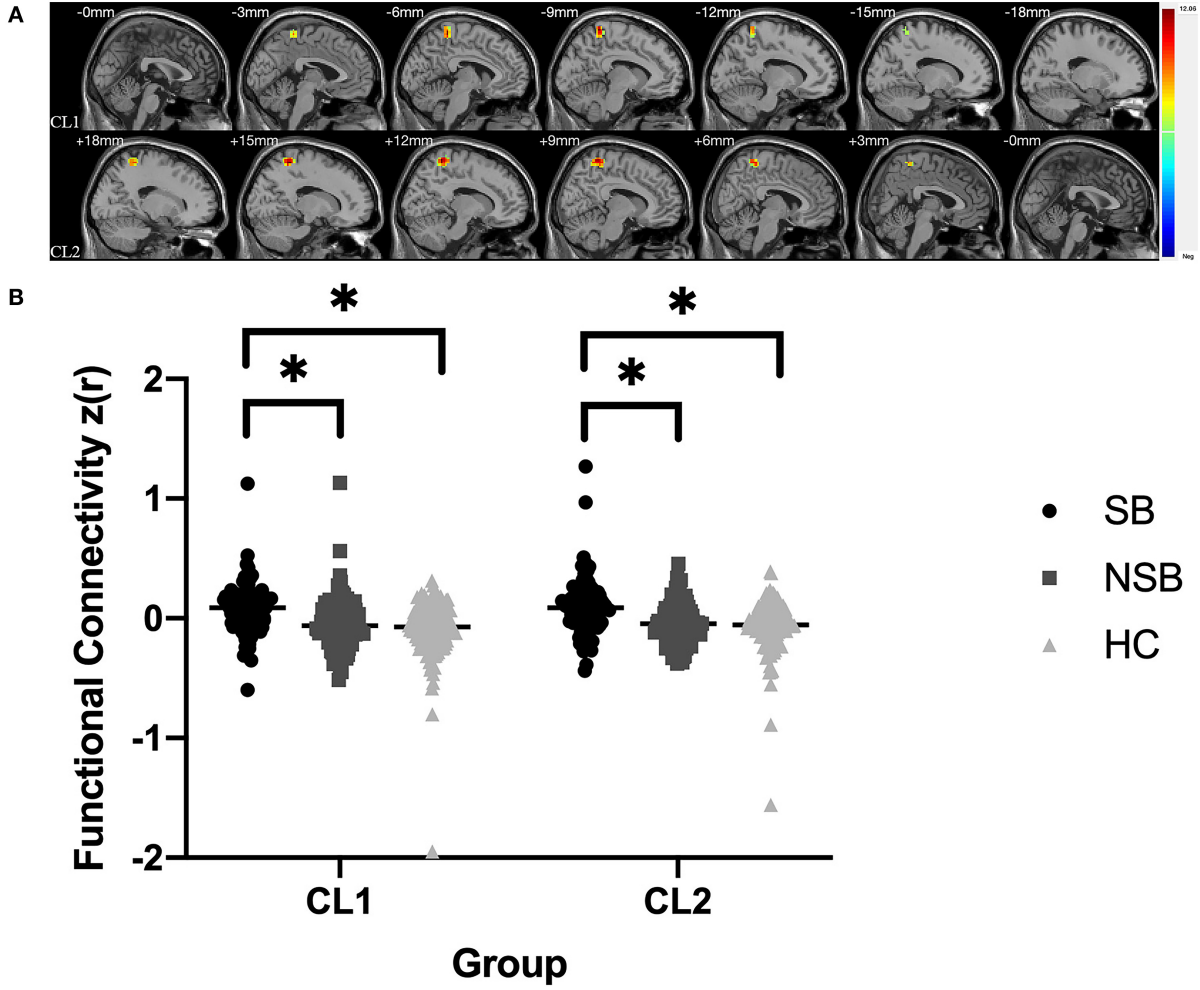
**(A)** Significantly different FC values between the right amygdala and bilateral paracentral lobule/precuneus in ROI-voxel analysis (GRF-corrected, *p* < 0.001); CL1: left paracentral lobule/precuneus; CL2: left paracentral lobule/precuneus. **(B)** *Post hoc* comparisons showing differences in FC values between pairwise groups (SB vs. NSB, SB vs. HC, NSB vs. HC); *Bonferroni-corrected, *p* < 0.017.

Figure 1 and Table 3 illustrates the significantly different connectivity of the key seed ROIs and all brain voxel among the three groups. ANCOVA showing abnormal FC values of the right amygdala with left paracentral lobule/precuneus (CL1) and right paracentral lobule/precuneus (CL2) in the three groups(GRF-corrected, *p* < 0.001; Figure 1A and Table 3). *Post hoc* tests revealed that the SB group showed significantly increased FC values between the right amygdala and bilateral paracentral lobule/precuneus compared to the NSB and HC groups (Bonferroni-corrected, *p* < 0.017; Figure 1B). Compared to the HC group, the NSB group exhibited no significant differences in FC values between the right amygdala and bilateral paracentral lobule/precuneus (Bonferroni-corrected, *p* > 0.017; Figure 1B).

**Table 3:** Clusters showing significant differences in FC among SB, NSB and HC groups.

**Index**	**Regions^**a**^**	**Cluster Size**	**Montreal Neurological Institute Coordinates**			**Mean FC values**			F^*^
			**x**	**y**	**z**	**SB**	**NSB**	**HC**	
CL 1	Right amygdala-left paracentral lobule/precuneus	62	−9	−42	72	0.07	−0.04	−0.09	13.26
CL 2	Right amygdala-right paracentral lobule/precuneus	121	12	−45	69	0.08	−0.05	−0.06	12.06

*CL, cluster*;

* *Significant at p < 0.001 corrected by GRF correction*.

a *AAL, Automated Anatomical Labeling*.

According to a one-way ANCOVA, we found no significant differences in the left amygdala-whole brain connectivity among the three groups (GRF-corrected, *p* > 0.001).

### Comparison of Connectivity Between SA and SI

**Figure 2:**
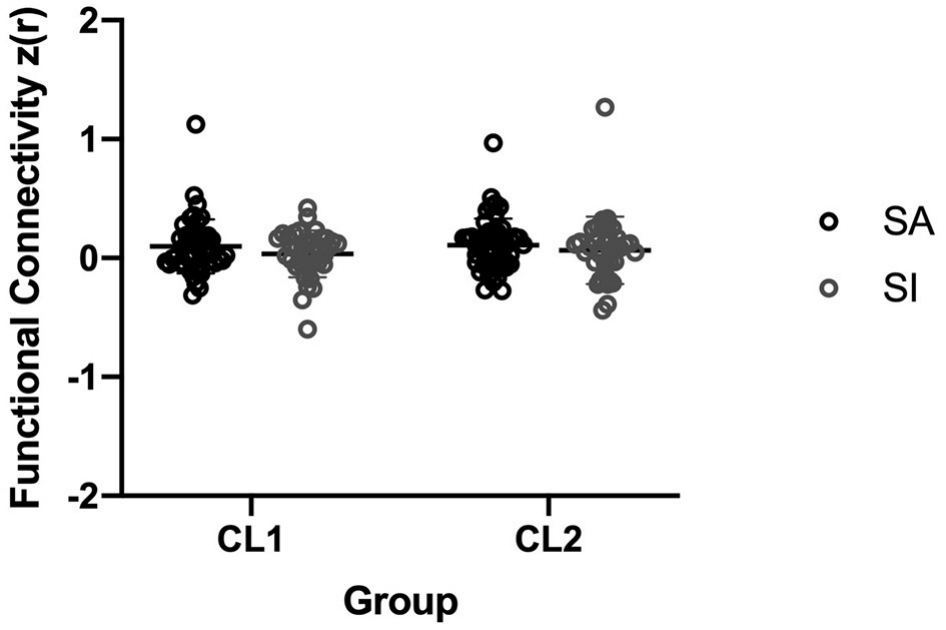
FC values comparison in the right amygdala-right paracentral lobule/precuneus between the SA and SI groups.

The results of additional analyses are presented in Figure 2. The FC values between the right amygdala and bilateral paracentral lobule/precuneus (CL1: *t* = 1.43, *p* = 0.16 > 0.05; CL2: *t* = 0.88, *p* = 0.38 > 0.05) showed no significant differences between the SA and SI group. However, there was a trend that the SA group exhibited higher FC values than the SI group.

### Correlation Between FC Values and Clinical Symptoms

To assess the correlation between FC values and clinical symptoms, we conducted a correlation analysis between the FC values of the significantly different regions according to the above mentioned ANCOVA and the HAMD scores and HAMA scores for the SB group. This analysis revealed no significant correlation in the FC values between the right amygdala and bilateral paracentral lobule/precuneus and clinical symptoms score for the SB group (*p* > 0.05).

### Additional Analysis

We reported the logistic regression analysis findings used to predict the relationship between suicidal behavior (yes or no) and age, sex, severity of depression and anxiety, and altered FC values. This analysis demonstrated that the FC values between right amygdala and bilateral paracentral lobule/precuneus, HAMD_17 scores were associate with suicidal behavior (Supplementary Table 1, Supplementary Material).

## Discussion

In the current study, we found that the FC values between the right amygdala and bilateral paracentral lobule/precuneus differed among the three groups. *Post hoc* analysis demonstrated that the SB group exhibited significantly increased FC values compared to the NSB and HC groups, but no significant differences were observed between the NSB and HC groups. No significant correlation was found in the correlation analysis between FC values and clinical symptoms for the SB group. Moreover, employing the two-sample *t*-test, we found no significant difference in the FC values between the SA group and the SI group, but the SA group exhibited higher FC values than the SI group. We also found that the FC values between the left amygdala and whole brain were not significantly different among the three groups.

We observed that the SB group showed increased FC values between the right amygdala and bilateral paracentral lobule/precuneus compared to the NSB and HC groups. Additionally, we did not observe any statistically correlation between altered FC values and clinical scales in a correlation analysis. However, we further conducted a logistic regression analysis regarding our data, the altered FC values of right amygdala-right paracentral lobule/precuneus and depression exhibited a significant correlation with suicidal behavior (Supplementary Material). This supports the concept that FC abnormalities between the right amygdala and paracentral lobule/precuneus are related to suicidal behavior and mood disorder, which is in keeping with the results of the previous study (Wei et al., 2018). The paracentral lobule and precuneus belong to the parietal lobe, which is involved in a variety of complex functions and is critical for somato-motor processing, mediation of subjective happiness (Sato et al., 2015), and emotion processing (Cavanna and Trimble, 2006). The amygdala is involved in several functions, including emotional responses and arousal. These functions all appear to be impaired in patients with suicide and mood disorder (Richard-Devantoy et al., 2014; Johnson et al., 2017). The results obtained in our study along with the proven psychological functions of these regions suggest that overprocessing of situational emotions and information is a severe defect that may lead to suicidal ideation or suicide attempts. Consistent with the present study, previous research has demonstrated that parietal abnormalities are related to suicidal behavior in mood disorder patients (Chen et al., 2015; Hibar et al., 2018). A recent fMRI study also indicated that suicide attempters exhibited higher brain activity in multiple regions, including the right inferior parietal lobe and left precuneus (Cao et al., 2015). These findings are in keeping with those obtained in this study. We speculated that increased brain connectivity might contribute to the risk of suicidal behaviors, which was reflected in the current study by the FC values abnormalities between the right amygdala and bilateral paracentral lobule/precuneus. However, these studies reached inconsistent conclusions and warrant further discussion. Several previous studies not only detected a greater activation but also found decreased activation in the cuneus and precuneus in patients with suicide attempts and mood disorder (Minzenberg et al., 2015). Future research designed to carefully characterize subtypes in these disorders may be better positioned to elucidate the relationship between the FC values in the parietal lobe and suicidal behaviors.

Previous studies have suggested that patients with suicide attempts differ biologically from those with suicidal ideation (Mann et al., 1999). In our study, we conducted a two-sample *t*-test to detect the difference between the SA group and the SI group. Although there was a trend that the SA group had higher FC values, we were unable to identify significant differences between the SA and SI group in the present study. Varying findings may be attributed to differences in sample size. This trend observed in additional analysis may still be important for understanding the development of suicidal ideation and attempts.

Another important finding is the brain lateralization of suicidality in the brain. In the current study, findings were limited to FC value abnormalities between the right amygdala and whole brain, and these abnormalities were not found between the left amygdala and whole brain. Interestingly, the right lateralization in patients with suicidal ideation was also reported in an earlier study (Caplan et al., 2010). However, a previous study observed left lateralization related to suicidal ideation in patients with MDD (Kim et al., 2017). One possible explanation for this apparent discrepancy is that participants in the previous study only had suicidal ideation or MDD, rather than suicide attempts or BD. Therefore, the findings should be carefully interpreted.

The present study discusses bilateral amygdala and whole-brain functional abnormalities in mood disorder patients with suicidal behavior in a cross-diagnostic sample (BD and MDD), but this study has several limitations. Firstly, the samples in this study combined patients with MDD and BD, who exhibit different biological changes. Although studies have noted that BD and MDD may share common biological mechanisms (Canali et al., 2015; Network Pathway Analysis Subgroup of Psychiatric Genomics Consortium, 2015; Gatt et al., 2015), the findings regarding the specificity of the diagnostic categories associated with suicide attempts warrant future investigation. Secondly, although we considered gender and age as covariances for statistical analysis, the possibility of gender and age bias should be noted. In the current study, women and middle-aged people are the majority; therefore, our results lack generalizability to other age and gender dominant groups. Thirdly, the method used for spatial normalization in the functional data processing is a commonly used template-based strategy that it could be complicated by signal dropout and low resolution in echo planar imaging (EPI) data. This supports the concept that FC abnormalities are related to suicidal behavior and mood disorder. Finally, the study was cross-sectional; therefore, it was difficult to acquire follow-up neuroimaging data determining whether promising biomarkers may predict suicide risk.

Despite these limitations, our study performed a cross-diagnostic investigation on the risk of suicide in patients with mood disorder, suggesting that suicidal behavior has its own neurological changes independent of these diagnostic categories. In particular, increased FC values between the right amygdala and paracentral lobule/precuneus may represent suicide-related brain activity alteration in patients with mood disorder.

## Data Availability Statement

The original contributions presented in the study are included in the article/Supplementary Material, further inquiries can be directed to the corresponding authors.

## Ethics Statement

The studies involving human participants were reviewed and approved by Institutional Review Board of China Medical University. Written informed consent to participate in this study was provided by the participants' legal guardian/next of kin.

## Author Contributions

RZ conceived, designed the study, and drafted the manuscript. LZ revised the manuscript and contributed to data analysis. SW contributed to drafting the manuscript. PW and XJ collected data. YT and FW supported data collection, coordinated the study, and revised the manuscript. All authors read and approved the final manuscript.

## Conflict of Interest

The authors declare that the research was conducted in the absence of any commercial or financial relationships that could be construed as a potential conflict of interest.
