# Supplementary material for: Increased Amygdala-Paracentral Lobule/Precuneus Functional Connectivity Associated With Patients With Mood Disorder and Suicidal Behavior
Source: Front Hum Neurosci. 2021 Jan 15;14:585664. doi: 10.3389/fnhum.2020.585664 (PMC7843440; doi:10.3389/fnhum.2020.585664)
Supplement: Supplementary file 1 [file Presentation_1.pdf]

Supplementary material

1. Binary Logistic Regression Analysis

Method

As major factors such as age, sex, severity of depression and anxiety, as well as the altered FC values might correlates our results, we validated our main findings using the analyses described below.

Binary logistic regression analysis was performed to predict a positive or negative suicidal behavior as the independent variable entering age, sex, severity of depression and anxiety(HAMD_17 scores, HAMA scores), FC values in significantly different clusters cross the three groups. Finally, it should be noted that the dependent variable measures the existence of suicidal behavior in mood disorder is YES, but not scale scores. YES is equal to 1 if the subject reported the suicidal behavior and 0 otherwise.

Result

According to the model in which only significant predictor was included, FC values in right amygdala-right paracentral lobule/precuneus and HAMD_17 scores were associated with suicidal behavior. None of the possible factors, including age, sex, severity of anxiety, FC values between right amygdala-left paracentral lobule/precuneus, had a significant predictor on the possibility of

suicidal behavior (Table S1).

Table S1 Logistic regression models in which patients with suicidal beahvior is predicted by FC values, severity of depression, anxiety, age, and sex.

Predictor	B	SE	P	OR	95%CI
CL 2	-3.68	0.89	0.001*	0.025	(0.004, 0.145)
HAMD_17 socres	-0.06	0.02	0.009*	0.945	(0.905, 0.986)

Abbrevation: CL 2, right amygdala-right paracentral lobule/precuneus; *P<0.001
